# Supplementary material for: Febuxostat enhances the anti-tumor efficacy of 2-fluoroadenine and 5’-methylthioadenosine in MTAP-deleted cancer
Source: bioRxiv. 2026 May 21:2026.05.19.726298. Preprint. [Version 1] doi: 10.64898/2026.05.19.726298 (PMC13228241; doi:10.64898/2026.05.19.726298)
Supplement: Supplement 1 — Supplemental Figure 1. Effect of XO and Febuxostat (FX) on 2FA toxicity at 48 h in two MTAP-deleted pancreatic carcinoma cell lines. N=4 per point. Supplemental Figure 2. Weight loss and toxicity in HT1080 SCID mice. A. Graph of Weight Changes. B. Representative H and E staining of the thymus, spleen, small intestine, and bone marrow (160x). Supplemental Figure 3. Analysis of the MiaPaCa-2 SCID experiment. A. Excised tumor weight. B. Individual mouse weight over time. C. Bone marrow H and E sections comparing vehicle- and high-dose 2FA-treated animals. Supplemental Figure 4. Analysis of the MiaPaCa-2 nude mice A. Excised tumor weight. B Image of excised tumors. C. Changes in mouse weight during the experiment. [file media-1.pdf]

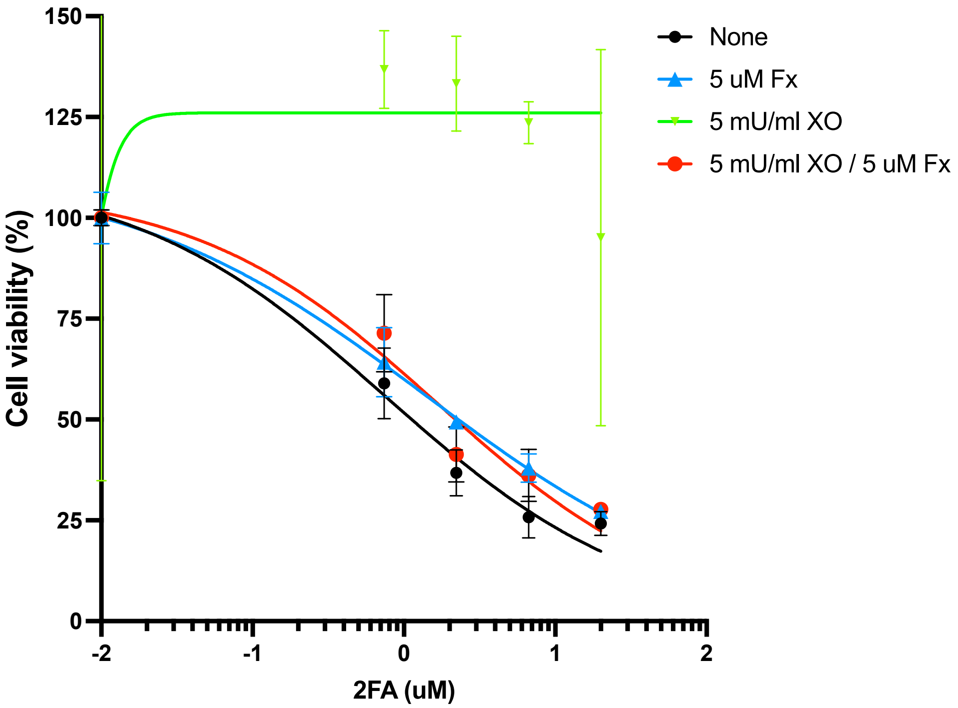


AsPC-1

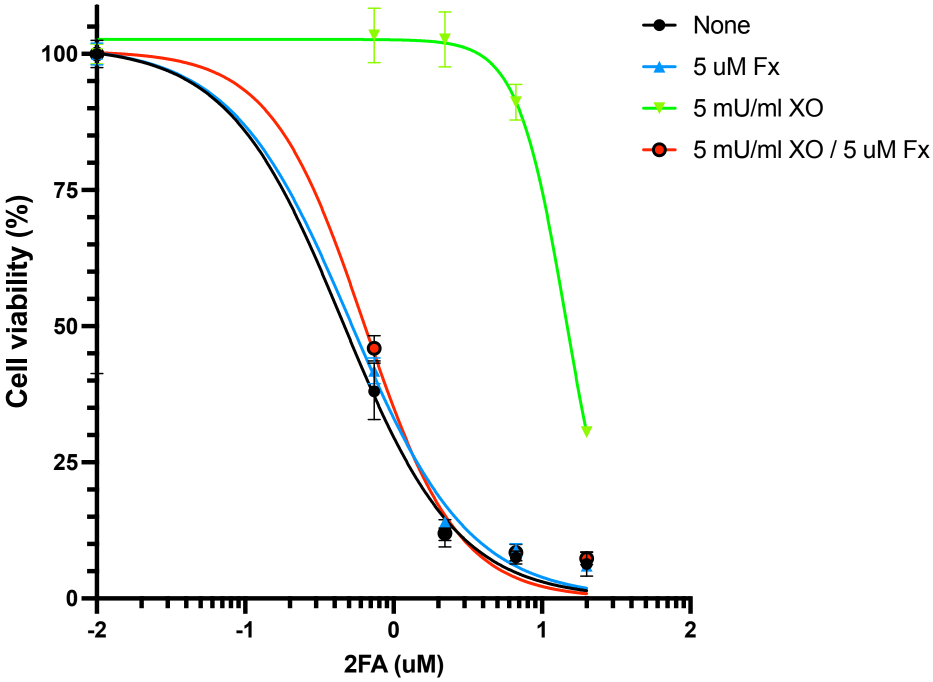


MiaPaca-2

Supplemental Figure 1

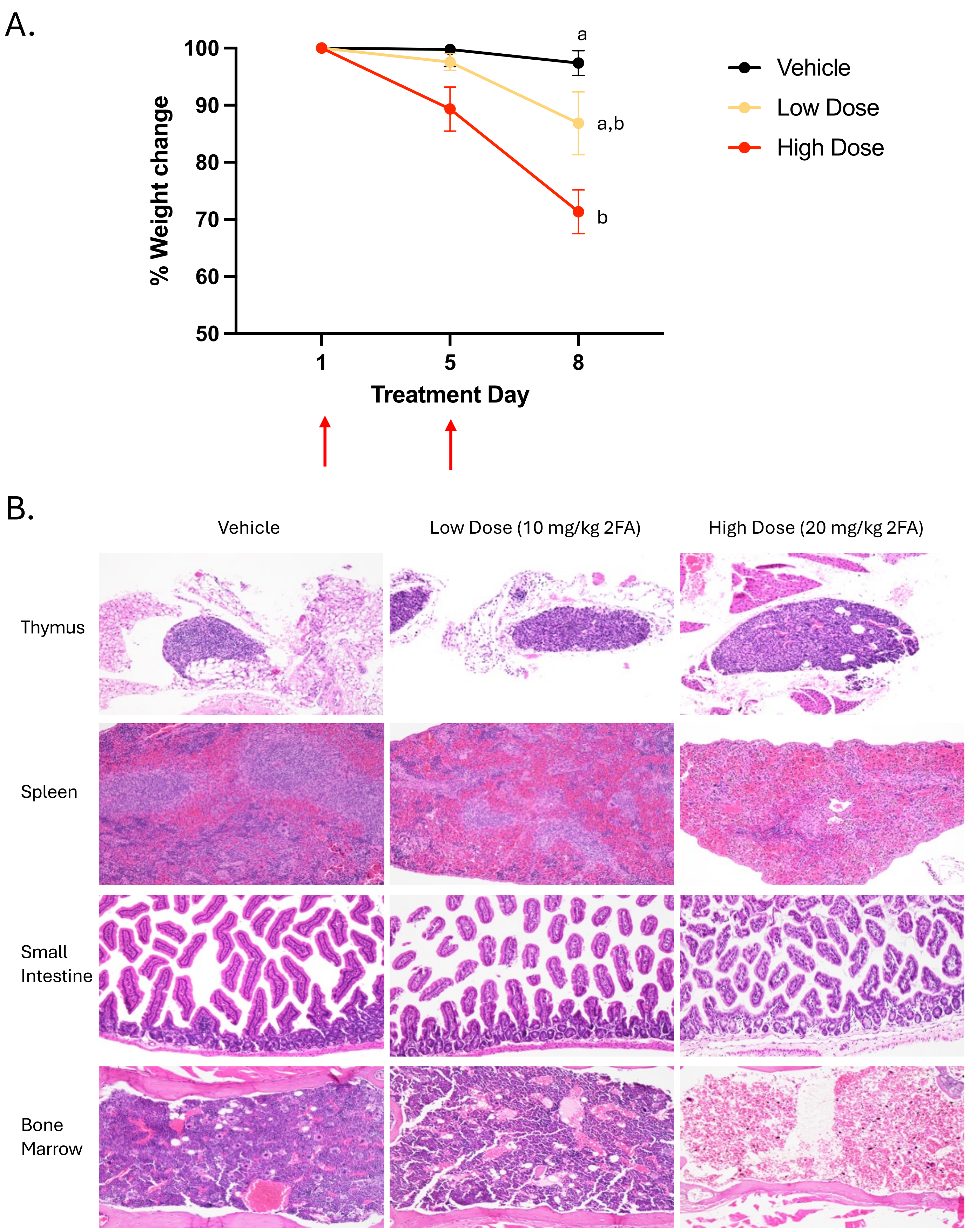


Supplemental Figure 2

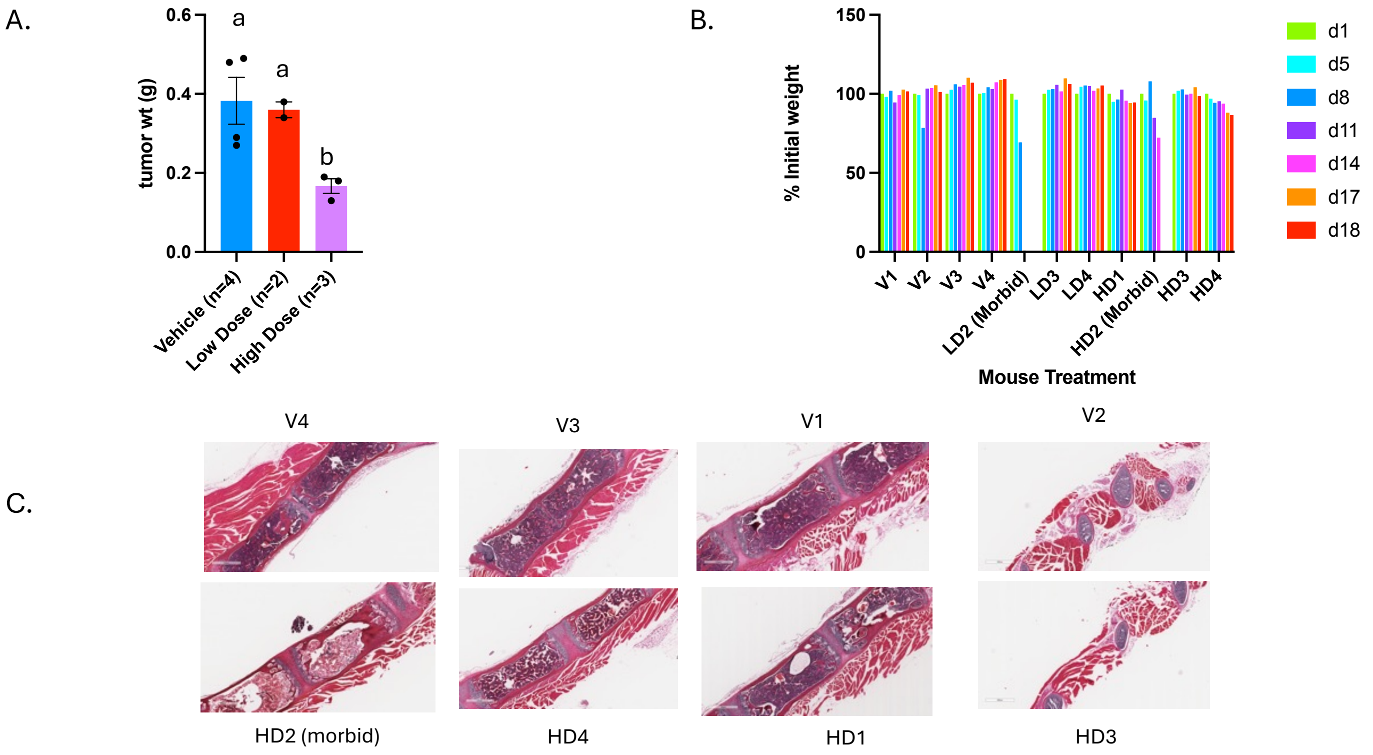


Supplemental Figure 3

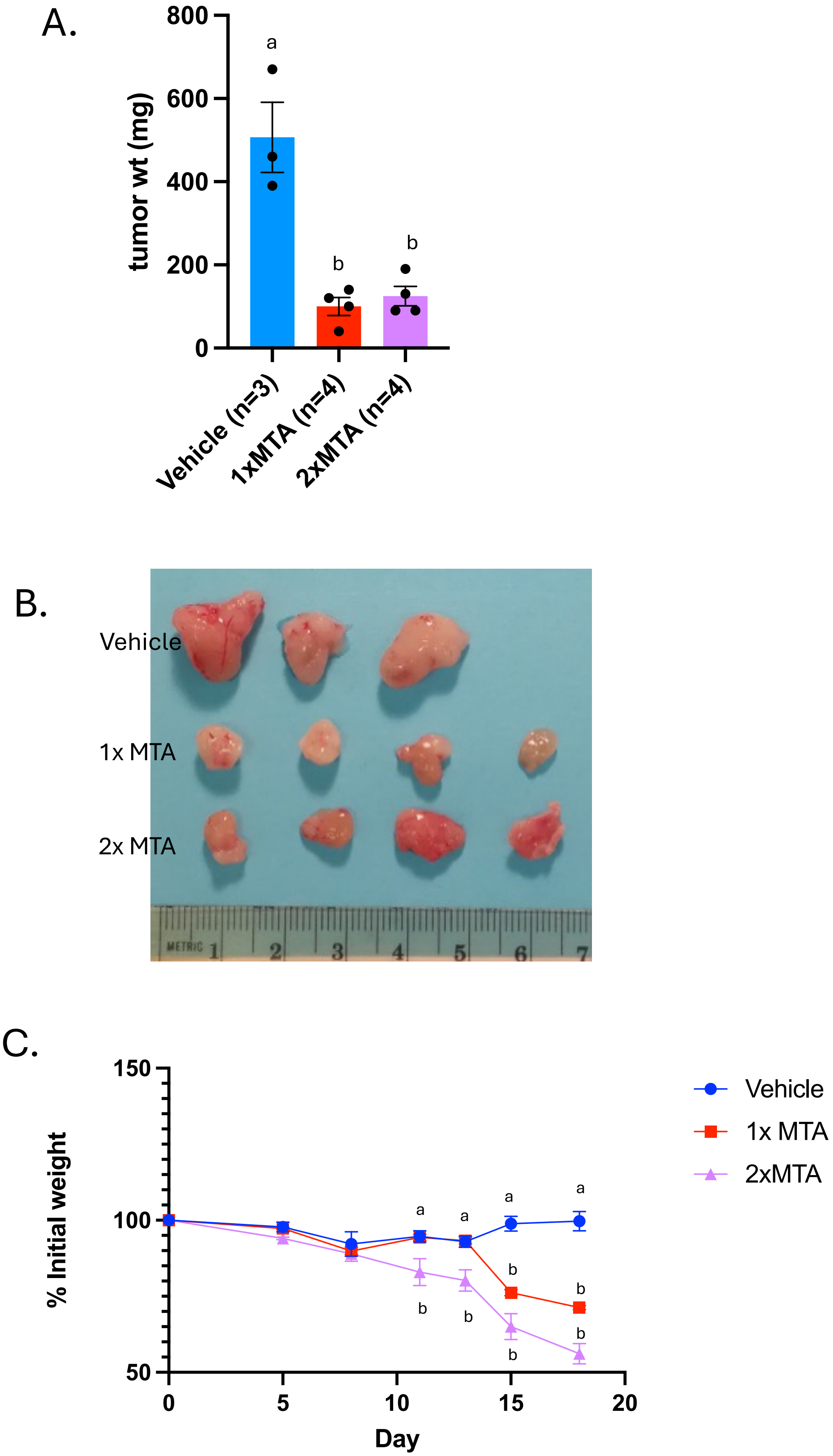


Supplemental Figure 4
